# Military environmental exposures and risk of breast cancer in active-duty personnel and veterans: a scoping review

**DOI:** 10.3389/fonc.2024.1356001

**Published:** 2024-03-13

**Authors:** Dylan J. Jester, Mehret T. Assefa, Daya K. Grewal, Abou M. Ibrahim-Biangoro, Jennifer S. Jennings, Maheen M. Adamson

**Affiliations:** ^1^ Women’s Operational Military Exposure Network Center of Excellence (WOMEN CoE), VA Palo Alto Health Care System, Palo Alto, CA, United States; ^2^ Department of Psychology, Palo Alto University, Palo Alto, CA, United States; ^3^ Department of Social and Behavioral Sciences, University of California, San Francisco, San Francisco, CA, United States; ^4^ War Related Illness and Injury Study Center (WRIISC), VA Palo Alto Health Care System, Palo Alto, CA, United States; ^5^ Department of Neurosurgery, Stanford University School of Medicine, Stanford, CA, United States

**Keywords:** female, breast neoplasm, women veterans, war, environmental epidemiology

## Abstract

**Background:**

The effects of military environmental exposures (MEE) such as volatile organic compounds (VOCs), endocrine-disrupting chemicals (EDCs), tactile herbicides, airborne hazards and open burn pits (AHOBP), and depleted uranium on health are salient concerns for service members and Veterans. However, little work has been done to investigate the relationship between MEE and risk of breast cancer.

**Data sources and methods:**

We conducted a scoping review on MEE, military deployment/service, and risk of breast cancer among active-duty service members and Veterans. PRISMA was used. PubMed, Embase, and citations of included articles were searched, resulting in 4,364 articles to screen: 28 articles were included.

**Results:**

Most papers on military deployment and military service found a lower/equivalent risk of breast cancer when comparing rates to those without deployment or civilians. Exposure to VOCs due to military occupation or contaminated groundwater was associated with a slightly higher risk of breast cancer. Exposure to Agent Orange was not associated with an increased risk of breast cancer. Evidence regarding EDCs was limited. No paper directly measured exposure to AHOBP or depleted uranium, but deployments with known exposures to AHOBP or depleted uranium were associated with an equivalent/lower risk of breast cancer.

**Conclusions:**

Women are the fastest growing population within the military, and breast cancer poses a unique risk to women Veterans who were affected by MEE during their service. Unfortunately, the literature on MEE and breast cancer is mixed and limited, in part due to the Healthy Soldier Paradox and poor classification of exposure(s).

## Introduction

The number of women Veterans served by the Department of Veterans Affairs - Veterans Health Administration (VA) more than quintupled between 2000 and 2021 (159,810 to 870,000+) (1, 2), while the number of men grew substantially slower over the same period (2, 3). In 2020, women comprised 19% of all military branches (2, 4), which highlights an ongoing need for the expansion of women-specific health services. The 2023 Office of Women’s Health - State of Reproductive Health governmental report found that abnormal breast conditions were reported as one of the top five reproductive and sexual health concerns for women Veterans aged 45+ (5). As VA projects the resources needed to care for the expanding women Veteran population, clinical and educational efforts must consider the unique health concerns faced by women Veterans.

Breast cancer (BC) is the most prevalent cancer among women, with around 300,000 cases diagnosed in the United States (U.S.) annually (6). One out of every eight women will be diagnosed at least once in their lifetime (6). The incidence rate (IR) of BC peaks in the 60s and 70s for women and the mortality rate increases exponentially with age (7), with Black women having the highest risk of mortality out of all racial and ethnic groups in the U.S. While the IR of BC has increased over the past two decades, the mortality rate has lowered substantially following advancements in early detection and treatment (7). Conversely, less than 1% of all BC patients are men (8) but BC in men is deadlier than in women (8). Military men with BC tend to present at a higher stage and with a larger tumor size than military women with BC, though demographics or tumor characteristics do not fully explain the higher rate of mortality in men with BC (9). BC is of great concern to VA and is a presumptive condition under The Sergeant First Class Heath Robinson Honoring our Promise to Address Comprehensive Toxics (PACT) Act of 2022. Presumptive conditions allow Veterans to receive care for ongoing health concerns that are of unknown etiology, and can be presumed to be related to service (10). Cancer of any kind remains an ongoing concern for Veterans as they age, and especially among Veterans with military environmental exposures (MEE).

The rates of cancers differ among active-duty personnel and the general U.S. population (11). Over 800 active-duty personnel receive a cancer diagnosis yearly, and tumor etiology is often correlated with service characteristics and MEE (12). These exposures include, but are not limited to, airborne hazards and open burn pits (AHOBP), asbestos, biological and chemical warfare tests, contaminated water, chemical agent resistant coating paint, embedded substances such as depleted uranium and lead, fuels, industrial solvents, ionizing radiation, mefloquine for malaria, nerve agents, noise, pesticides, perfluoroalkyl and polyfluoroalkyl substances, pyridostigmine bromide pills for sarin gas exposure, tactile herbicides, and vaccines. Cancer among current and former military personnel with known MEE persists as a complex health concern (12–15).

The current literature on MEE and cancer is limited. For example, the tactile herbicide Agent Orange was linked to an increased incidence of several cancers, including leukemia and cancers that start in soft tissues (16), and a slightly higher rate of BC was found among military personnel when compared to civilians (17). However, higher rates of BC may be tied to confounding risk factors in military personnel, such as delayed age of first childbirth or increased use of contraceptives. Additionally, military personnel often have greater access to routine screening, resulting in quicker identification of early-stage BCs (18, 19). In other words, tying BC incidence to MEE rather than characteristics associated with service (i.e., confounding factors) is a difficult task.

Combat exposure has increased from 7% to 24% when comparing pre-1990 to post-1990 women Veterans, suggesting that MEE concerns may grow among women Veterans in the coming decades (20). However, few have investigated BC in association with specific MEE. Therefore, we conducted a scoping review to determine whether deployment/military service and MEE affect the risk of BC among active-duty personnel and Veterans.

## Methods

### Search strategy

**Figure 1:**
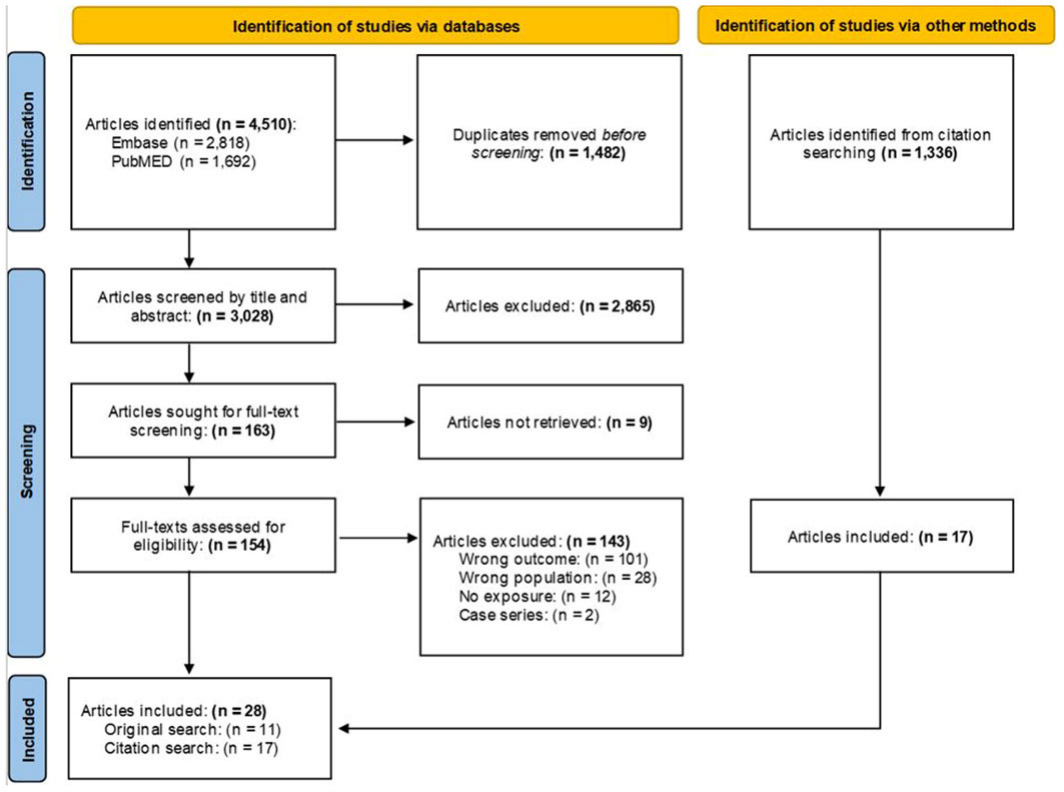
PRISMA flow chart. MeSH Trees used: Diseases Category ==> Neoplasms ==> Neoplasms by Site ==> Breast Neoplasms. Titles and abstracts were searched using the terms: (“breast cancer*” OR “breast neoplasm*” OR “breast tumor*” OR “breast metas*” OR “mammary metas*” OR “mammary cancer*” OR “malignant neoplasm* of the breast*” OR “malignant neoplasm* of breast*” OR “breast malignant neoplasm*” OR “malignant tumor* of breast*” OR “malignant tumor* of the breast*” OR “breast malignant tumor*” OR “cancer of breast*” OR “cancer of the breast*” OR “mammary carcinoma*” OR “mammary neoplasm*” OR “breast carcinoma*” OR “mastect*” OR “lumpect*” OR “mammogr*”) AND (Veteran* OR military OR combat OR deploy* OR undeploy* OR soldier* OR war OR wars OR warzone OR “department of defense” OR DOD OR front-line* OR duty OR enlist*). Asterisk wildcards were used to find word endings. Terms were left purposefully broad to examine the largest possible selection of the literature. Prospective or retrospective cohort, case-control, cross-sectional, ecological, or related study designs (e.g., case-cohort) were included. Case studies, case series, reviews and meta-analyses, book chapters, theses, and dissertations were excluded.

Unlike systematic reviews that focus on a specific research question, scoping reviews ask broad research questions to characterize and understand a developing and heterogenous area within the literature (21). Search terms were compiled using PubMed’s Medical Subject Headings (MeSH) trees and through consultation with the California War Related Illness and Injury Study Center (CA WRIISC), the Women’s Operational Military Exposure Network Center of Excellence (WOMEN CoE) and Advisory Board, and staff oncologists at VA Palo Alto Health Care System. Relevant articles were searched for in PubMed and Embase and terms can be found in the notes of the Preferred Reporting Items for Systematic Reviews and Meta-Analyses (PRISMA) flowchart (**Figure 1**). Some articles broadly examined cancer incidence and did not mention BC in the title or abstract, but included estimates of BC within the results/tables. Therefore, four authors (AIB, DJJ, DKG, MTA) screened citations from the included articles to find these additional manuscripts.

### Inclusion criteria

To be included, studies had to: (1) enroll active-duty personnel, Reservists, or Veterans, (2) measure MEE or military service/deployment, (3) concern BC risk (i.e., papers on BC mortality were excluded), and (4) have an English full-text.

### Study selection

Covidence software was used to collate and screen the articles. Four authors (AIB, DJJ, DKG, MTA) screened titles/abstracts and full-texts and met weekly to resolve disagreements through discussion. The database search was conducted on June 16, 2023, and resulted in a total of 4,510 articles. After the removal of 1,482 duplicates, 3,028 titles and abstracts were screened and 2,865 were excluded. A total of 163 full-texts were assessed, of which 11 were included. After screening an additional 1,336 citations from the included articles, 17 were retained for a final total of 28 articles.

### Data extraction

Data extraction was completed by four authors (AIB, DJJ, DKG, MTA) with each paper receiving at least two checks for accuracy and included the following headings: author/publication year, sample characteristics, sample size, exposure, results, warfare era/service years, and diagnosis years. See **Table 1** for the characteristics of each study.

**Table 1:** Characteristics of the included studies.

Study Name (Year)	Sample Characteristics (Country/Region)	Sample Size	Exposure	Results	Era/ Service Years	Diagnosis Years
Ajene et al. (2004) (22)	Navy active-duty personnel (U.S.)	78 women	Military Service (Various Periods)	For women, breast cancer was observed at a rate of 8.5 cases per 100,000 personnel, with a rate of 56.4 cases per 100,000 personnel seen in the 40+ age group. The authors state that their rate was much lower than historical Navy rates (34.1 per 100,000) and Surveillance, Epidemiology, and End Results (SEER) rates (143.2 per 100,000), likely due to the younger age of the sample.	N/A	1998-2000
Armed Forces Health Surveillance Center (2013)	Active-duty personnel (U.S.)	All women in active component of Armed Forces with any service from 2000-2012	Military Service (Various Periods)	Between 2000 and 2012, 1,092 women were diagnosed with breast cancer. The overall crude incidence rate was 40.6 per 100,000 person-years. The annual incidence rate was lowest in 2006 at 28.6 per 100,000 person-years and highest in 2001 at 53.6 per 100,000 person-years. Active-duty women who served in the Air Force (IRR=2.4), identified as non-Hispanic Black (IRR=2.2), were older (40+) (IRR=27.1), senior officers (IRR=4.1), and women serving in healthcare roles (IRR=2.1) or administrative/supply roles (IRR = 1.6) had an increased risk of breast cancer. Women who served in the Marine Corps (reference group), identified as Hispanic (reference group), were younger women (<25) (reference group), were junior enlisted (reference group), and women with “other” duties (reference group) had a decreased risk of breast cancer. Women with combat-specific duties had a marginally increased risk of breast cancer (IRR = 1.1) when compared to women with “other” duties.	N/A	2000-2012
Bytnar et al. (2023) (23)	Active-duty personnel and civilians (U.S.)	Several million (not stated)	Military Service (Various Periods)	No significant increased risk of breast cancer was found among active-duty personnel compared to the general population, and this did not differ by race: White IRR=1.06 [0.98, 1.13]; Black IRR=1.06 [0.96, 1.16] women service members. When stratified by age, Black (IRR=0.98 [0.86, 1.12]) and White (IRR=0.96 [0.85, 1.07]) women military service members aged 20-39 had no significant increased risk of breast cancer when compared to the general population. However, Black (IRR=1.17 [1.01, 1.34]) and White (IRR=1.15 [1.04, 1.26]) women military service members aged 40-59 had a statistically increased risk of breast cancer when compared to the general population. Further stratification by cancer stage (local, regional, and distant) showed only a significant age effect for local breast cancers (confined to the breast), but not for regional (extends to the surrounding lymph nodes, organs, or tissues) or distant cancers (extends to distant organs or lymph nodes).	N/A	1990-2013
Carran et al. (2012) (24)	Adult children of Veterans (New Zealand)	76 adult children of New Zealand Veterans	Dibutylphthalate was applied daily to soldiers’ clothing as an acaricide during the Malayan Emergency	Authors found a slightly increased risk of breast cancer among female adult children of New Zealand Veterans deployed to Malayasia who were exposed to the endocrine-disrupting chemical dibutylphthalate. However, results were based on 3 incident cases.	Malayan Emergency: 1948-1960	N/A
Gaffey et al. (2023) (25)	Veterans (U.S.)	576,601 women, 24.6% (n=141,935) deployed	Military Service (Post-9/11 conflicts)	Those who deployed in support of OEF/OIF were 23% [14%, 27%] less likely to receive a breast cancer diagnosis than women who did not deploy after adjusting for age, race and ethnicity, marital status, military service connection, smoking status, body mass index, history of alcohol use disorder, hormonal contraceptive use, and hormone replacement therapy use. IRs were 34 and 44 per 100,000 person-years for OEF/OIF-deployed Veterans and for those not deployed in support of OEF/OIF, respectively.	OEF/OIF	2001-2021
Hansen et al. (2012) (26)	Military personnel (Denmark)	218 cases of breast cancer 899 age-matched controls	Military night shift work, leisure time sun exposure, and diurnal preference	Women with any history of night shift work exhibited an increased odds of breast cancer (OR: 1.4 [0.9, 2.1]) compared to those who never worked night shifts. Breast cancer risk increased with longer duration of night shift work and cumulative number of shifts, but risk was neutral for those working fewer than three-night shifts per week. Women with the highest tertile of cumulative night shift exposure had an increased odds of breast cancer (OR=2.3 [1.2, 4.6]). Women with a morning chronotype preference (natural inclination to be more active during the morning) and intense night shifts had the largest risk (OR=3.9 [1.6, 9.5]). These findings persisted after adjusting for age, hormone replacement therapy, number of childbirths, age at menarche, years of education, sunbathing frequency, and smoking status.	N/A	1990-2003
Hoiberg & Ernst (1980) (27)	Navy active-duty personnel (U.S.)	364 women officers and enlisted personnel	Military Service (Various Periods)	The overall breast cancer incidence rate among women was 34.1 per 100,000 people, with rates increasing with age. The highest rate was among women 46+ years old at 496.3 per 100,000 people. Rates by Age: 17-25: 1.0, 26-35: 33.0, 36-45: 262.2, 46+: 496.3 per 100,000	1966-1976	1965-1976
Kang et al. (2000) (28)	Veterans (U.S.)	6430 women Veterans: 3,393 Vietnam War Veterans 3,038 non-Vietnam War Veterans	Military Service (Vietnam War)	Breast cancer was reported in 5% of Vietnam Veterans and 4.1% of non-Vietnam Veterans. The crude and adjusted odds of developing breast cancer were not statistically different between the two Veteran groups (Crude OR=1.22 [0.96, 1.55]; Adjusted OR=1.18 [0.91, 1.51]) controlling for age, race, branch, pay grade, marital status, nursing occupation, smoking, alcohol consumption, family history, use of oral contraceptives, and use of postmenopausal estrogen or progestin use. Risk of breast cancer increased with age.	Vietnam War	N/A
Katuwal et al. (2018) (29)	Military personnel and civilians (Finland, Sweden, Norway, Denmark, Iceland)	7.5 million adults	Military Service (Various Periods)	26 cases of breast cancer were reported among military personnel from four out of the five Nordic countries. Of the 54 occupational categories, military personnel had the highest overall risk for breast cancer (SIR: 1.58 [1.03, 2.32]). SIR was also provided by histology (ductal and lobular breast cancer) and country, with highest SIR observed for ductal breast cancer (SIR=1.41 [0.75, 2.42]) and Denmark (SIR=2.14 [0.70, 5.01]).	1961-2005 divided into three periods: 1961-1975 1976-1990 1991-2005	1961-2005
Lee et al. (2023) (30)	Veterans (Korea)	1,301,331 Korean Vietnam War Veterans	Military Service (Vietnam War)	A total of 123 new cases of breast cancer were identified among Korean Vietnam War Veterans. The breast cancer incidence rate was 5.1 per 100,000 person-years; however, the SIR was not significantly elevated (SIR=1.05 [0.88, 1.26]).	Vietnam War	2002-2020
Lee et al. (2016) (31)	Active-duty personnel (U.S.)	All individuals in active component of Armed Forces with any service from 2005-2014	Military Service (Post-9/11 conflicts)	652 cases of breast cancer were observed (IR=31.8 per 100,000). Active-duty women who identified as non-Hispanic Black (Risk Ratio [RR]=1.29), were officers (RR=2.73), had a healthcare occupation within the military (RR=1.52), and were older (20-24 as reference: 25-29 RR=3.29; 30-34 RR=9.65; 35-39 RR=22.32; 40+ RR=59.18) had an increased risk of breast cancer.	July 1, 2005-December 31, 2014	N/A
Macfarlane et al. (2003) (32)	Veterans (United Kingdom)	51,721 Gulf War Veterans and 50,755 service personnel	Military Service (Gulf War)	Non-Gulf War service personnel were matched for age, sex, rank, service, and level of fitness. A total of 6 occurrences of breast cancer in Gulf War cohort and 10 in the non-Gulf War cohort were identified. Breast cancer risk did not differ between the cohorts (IRR=0.59 [0.21, 1.62]). There was no change in the IRR after adjusting for smoking behavior and alcohol consumption.	Gulf War	1991-2002
Mahar et al. (2022) (33)	Military Veterans and Royal Police Veterans (Canada)	30,576 Veterans 122,293 matched general population	Military Service (Gulf War & Post-9/11 conflicts)	The incidence rate of breast cancer in women Veterans was 30.01 [18.91, 47.63]) per 100,000 person-years, compared to the general population (25.06 [19.46, 32.27] per 100,000 person-years (matched on age, sex, residential geography, and community socioeconomic status). Women Veterans had no statistically significant increased risk of breast cancer when compared to the general population, before or after adjusting for the matching variables (Crude HR=1.20 [0.71, 2.03]; Adjusted HR=1.19 [0.70, 2.02]).	Gulf War and Post 9/11 conflicts: April 1, 1990-December 31, 2018	Baseline health insurance date following military service – 2019
Mohr et al. (2013) (34)	Active-duty personnel (U.S.)	600 incident cases of breast cancer 600 controls	25-hydroxyvitamin D (25(OH)D)	In the adjusted models, no statistically significant relationship was found between serum Vitamin D levels and odds of breast cancer. Inverse trends were present among women with a blood draw within 90 days of their breast cancer diagnosis, where women in the lowest quintile of 25(OH)D had a higher estimated risk of breast cancer (OR=3.3 [1.6, 7.1]) compared to women in the highest quintile. It was not made clear by the authors if Vitamin D levels were affected by fortified foods or supplements in the sample.	1994-2009	N/A
Rennix et al. (2005) (35)	Army active-duty personnel (U.S.)	274,596 women Army personnel	Volatile Organic Compounds (VOCs)	184 cases of invasive breast cancer were identified. Incidence of breast cancer was significantly elevated in women ages 17-34 years, especially among Black women, when compared to general population. Women in occupations with medium or high potential exposure to VOCs (e.g., chlorinated hydrocarbons, aromatics, alcohols, aldehydes, ketones, other solvents and distillates) had an IRR of 1.48 [1.01, 2.07], adjusting for race, age at diagnosis, and year of diagnosis. Enlisted Black women had higher IRR compared to enlisted White women (IRR=1.43 [1.01, 2.07]) and IRR increased with age at diagnosis (IRR=2.17 [1.98, 2.39]) and year of diagnosis (IRR=1.24 [1.11, 1.39]) (I.e., more recent time-periods had an increased risk compared to older time-periods).	Pre-Gulf War & Gulf War: 1980-1996	1980-1996
Ruckart et al. (2015) (36)	Marines stationed at Camp Lejeune (U.S.)	71 male breast cancer cases 373 controls	VOC-contaminated drinking water	The odds for breast cancer among ever being stationed at Camp Lejeune was 1.14 [0.65, 1.97]. Adjusted ORs for high residential cumulative exposures to tetrachloroethylene, t-1,2 dichloroethylene, and vinyl chloride were 1.20 [0.16, 5.89], 1.50 [0.30, 6.11], and 1.19 [0.16, 5.89], respectively, with a monotonic exposure response relationship for PCE only. Ever being stationed at Camp Lejeune and high cumulative exposures to VOCs were associated with an earlier age of onset for male breast cancer, but confidence intervals were wide due to the small sample size.	Camp Lejeune Garrison Exposure: 1953-1987	2004-2012
Storm et al. (2006) (37)	Balkan Veterans (Denmark)	460 women	Military Service (Balkan Conflicts)	Among military women, there were 3 observed cases and no statistically significant increased risk of breast cancer (SIR=1.5 [0.3, 4.3]). The authors suggested that exposure to depleted uranium was a major concern during these conflicts, though it was not directly measured.	Balkan war (January 1, 1992-December 31, 2001)	1992-2002
Strand et al. (2014) (38)	Military peacekeepers (Norway)	268 women	Military Service (Kosovo)	Of the 2 cancers observed in women, 1 was breast cancer for a combined SIR of 0.55 [0.07, 1.98].	First Gulf War and the Balkans conflict	1999-2011
Strand et al. (2015) (39)	Military peacekeepers (Norway)	21,582 military peacekeepers	Military Service (Lebanon)	No military peacekeeper was diagnosed with breast cancer (SIR=0.00 [0.00, 2.07]).	Israel-Lebanon war (1978-1998)	1978-2012
Strand et al. (2020) (40)	Military peacekeepers (Norway)	275 women	Military Service (Kosovo)	Of the 8 cancers observed in women, 3 were cases of breast cancer (all-site cancer SIR=1.10 [0.47, 2.16]). There was 1 case of breast cancer observed in men (SIR=6.00 [0.15, 33.4]).	Bosnian war/Balkan conflict	1999-2016
Yamane et al. (2006) (41)	Air Force active-duty personnel (U.S.)	76,477 women	Military Service in the U.S. Air Force (Gulf War & Post-9/11 conflicts)	Breast cancer was listed as the most frequent cancer among women in the Air Force (26.7% of all cancers in women), but service members did not have a statistically increased risk (SIR=0.88 [0.76, 1.01]).	1989-2002	1989-2002
Yi (2013) (42)	Veterans (Korea)	185,265 men	Military Service (Vietnam War)	There were 8 observed cases of breast cancer, but service members did not have a statistically increased risk (SIR=1.37 [0.67, 2.83]).	Vietnam War	1992-2003
Yi & Ohrr (2014) (43)	Veterans (Korea)	180,251 Veterans	Agent Orange	Vietnam-era Veterans exposed to high levels of Agent Orange did not have an increased risk of breast cancer (Adjusted HR=0.53 [0.12, 2.26]). The authors did not specify the number of women Veterans in the study, though it is presumed to include very few, if any, given the cohort characteristics from a previous publication (Yi, 2013) (42).	Vietnam War	1992-2003
Young et al. (2010) (44)	Veterans (U.S.)	621,902 Gulf War Veterans (43,533 women)	Military Deployment	Compared to non-Gulf War Veterans, Gulf War Veterans had a statistically equivalent incidence rate of breast cancer among men (PIR=0.78 [0.39, 1.58]) and women (PIR=1.01 [0.86, 1.20]). These rates did not change appreciably when restricting the sample to Gulf War Army or Gulf War Marine Corps members.	Gulf War	1991-2006
Zhu et al. (2009) (17)	Active-duty personnel and civilians (U.S.)	Several million (not stated)	Military Service (Gulf War & Post-9/11 conflicts)	Breast cancer was the most common cancer among active-duty military women (n=864). The authors found a slightly higher IRR for Black military women 1.37 [1.21, 1.55] and for White military women 1.19 [1.09, 1.30] when compared to civilians. When examining diagnosis year (1990-1994 to 2000-2004), breast cancer incidence did not statistically differ for the military population.	Gulf War & Post-9/11 conflicts	1990-2004
Zullig et al. (2012) (45)	Veterans (U.S.)	4,875,740 Veterans 31,010 incident cancers	Military Service (Various Periods)	Among women Veterans, breast cancer was the most diagnosed cancer, accounting for 29.5% of all female cancers. Among men, breast cancer accounted for 0.2% of all male cancers.	N/A	2007
Zullig et al. (2017) (46)	Veterans (U.S.)	5,894,299 Veterans 46,166 incident cancers	Military Service (Various Periods)	Among women Veterans, breast cancer was 30.23% of all female cancers. Among men, breast cancer accounted for 0.17% of all male cancers. Results did not change appreciably from the Zullig et al. (45) findings, although breast cancer became a slightly higher proportion of all cancer cases among Black (1.26%) and “Other Minority” (1.58%) Veteran groups when compared to White Veterans (0.96%). This difference could be explained by the increase of Asian, Black, Hispanic, and Native/Indigenous women in the military.	N/A	2010
Zullig et al. (2019) (47)	Veterans (U.S.)	1,330 women diagnosed with invasive cancer	Military Service (Various Periods)	Breast cancer was the most diagnosed cancer (approximately 30%) among women Veterans with an invasive cancer diagnosis. A total of 402 breast cancer cases were identified, which did not change appreciably by race. Most women Veterans presented with an early stage of breast cancer (50% Stage 1; 32% Stage 2; 14% Stage 3; 4% Stage 4), unlike women Veterans diagnosed with lung and bronchus (27% Stage 3; 35% Stage 4) and colorectal cancers (23% Stage 3; 21% Stage 4).	N/A	2010

APR, Adjusted Proportional Incidence Ratio; HR, Hazard Ratio; IRR, Incidence Rate Ratio; PIR, Proportional Incidence Rate; SIR, Standardized Incidence Ratio; OR, Odds Ratio; OEF, Operation Enduring Freedom; OIF, Operation Iraqi Freedom; U.S., United States of America; N/A, Not Available.

The sample for which breast cancer outcomes are reported are included in the sample size column of **Table 1**. For papers that provided outcomes for both men and women, the full sample size was included. For papers that examined breast cancer exclusively in either men or women, only the corresponding sample size was included (vs the entire sample).

## Results

In total, 28 papers were synthesized. Sample size ranged from 64 to millions. Several military conflicts were included: Malayan Emergency, Vietnam War, Israel-Lebanon Conflicts, Persian Gulf War, Kosovo War, Bosnian War, Croatian War of Independence, and post-9/11 conflicts (Operation Enduring Freedom [OEF], Operation Iraqi Freedom [OIF], Operation New Dawn [OND]). More than half of the studies used a case-control or cohort study design. Several MEE were examined: military service/deployment, volatile organic compounds (VOCs), endocrine-disrupting chemicals (EDCs), Agent Orange, and ultraviolet B radiation (Vitamin D synthesis).

### Military service/deployment

Twenty-three papers measured BC among military personnel and those deployed to specific conflicts (17, 22, 23, 25–33, 37–42, 44–48).

#### Risk compared to civilians & standardized rates

Nine papers compared risk of BC in military personnel compared to civilians or standardized national rates. Zhu and colleagues (2009) conducted a cohort study and compared military and civilian cancer surveillance data. They found a slightly higher IRR for Black military women 1.37 [1.21, 1.55] and for White military women 1.19 [1.09, 1.30] when compared to civilians (17). Katuwal and colleagues (2018) carried out a cohort study of nearly 7.5 million Nordic women from 1961-2005 and found roughly 375,000 cases of BC. Military personnel had the greatest SIR for BC at 1.58 [1.03, 2.32] (29). Storm and colleagues (2006) followed 460 women military personnel who deployed to the Balkans and found no significantly increased risk (Standardized Incidence Ratio [SIR]=1.5 [0.3, 4.3]) (37). Yamane and colleagues (2006) compared BC IRs among 76,477 U.S. Air Force active-duty personnel to national IRs, and found the rates to be statistically equivalent (SIR=0.88 [0.76, 1.01]) (41). Yi (42) included 185,265 male Vietnam Veterans from Korea (n=8 cases), but found a statistically equivalent rate of BC when compared to the general population (SIR=1.37 [0.67, 2.83]) (42). Strand and colleagues (2015) conducted a cohort study of 21,582 Norwegian male military peacekeepers deployed to Lebanon and zero cases of BC were observed (SIR=0.00 [0.00, 2.07]) (39). Mahar and colleagues (2022) published a cohort study of 30,576 Canadian Veterans and police and 122,293 matched controls. Women Veterans had no statistically significant increased risk of BC (adjusted HR=1.19 [0.70, 2.02]) (33). Bytnar and colleagues (23) reconducted the analysis from Zhu et al. (17) using military (n=1,185 cases) and civilian (n=183,042 cases) cancer surveillance data. Black (IRR=1.06 [0.96, 1.16]) and White (IRR=1.06 [0.98, 1.13]) women military personnel had no significant increased risk of BC when compared to the general population (23). Lee and colleagues (2023) compared 250,842 Vietnam-era Korean Veterans (353 women) with 1,050,489 matched Korean civilians (1,695 women) and observed 123 cases of BC (IR=5.1 [4.2, 6.0]), but found no significantly increased rate (SIR=1.05 [0.88, 1.26]) (30).

#### Demographics & work characteristics

Six papers provided estimates that differed by demographic or work characteristics. Hansen and Lassen (26) studied 218 women with BC and 899 age-matched controls from a nested cohort study of 18,551 women Danish military employees. Women with the highest tertile of cumulative night shift military work exposure had an increased odds of BC (OR=2.3 [1.2, 4.6]) and women with a morning chronotype preference (inclination to be more active during the morning) and intense night shifts had the largest risk (OR=3.9 [1.6, 9.5]) (26). The Armed Forces Health Surveillance Center (2013) published a cohort study and found that the IR of BC among active-duty service women was 40.6 per 100,000 from 2000-2012 (48). Non-Hispanic Black women, older women, senior officers, and women serving in healthcare or administrative roles had an increased risk of BC (48), and women with combat-specific duties had a mildly increased risk. Conversely, women who served in the U.S. Marine Corps, those who identified as Hispanic, younger women, junior enlistees, and women with “other” duties had a decreased risk of BC (48). Lee and colleagues (2016) examined all U.S. active-duty personnel from 2005-2014, of which 652 cases of BC were observed (IR=31.8 per 100,000). Active-duty women who were non-Hispanic Black, officers, healthcare workers, or older had an increased risk of BC (31). Zullig and colleagues (2012) found no major differences in BC incident diagnoses among Black, “Other,” and White Veterans from the 2007 Veterans Affairs Central Cancer Registry (45). Zullig and colleagues (2017) updated their 2007 analysis (Zullig et al. (45),) with data from 2010, but results did not change appreciably (46). Zullig and colleagues (2019) conducted a cross-sectional study of 1,330 incident invasive cancer cases among women Veterans in 2010; BC was the most common invasive cancer (30.23%, n=402), but it did not differ by race (47).

#### Deployment characteristics

Four papers compared deployment characteristics. Kang and colleagues (2000) followed 3,392 Vietnam-era deployed women Veterans (n=170 cases) and 3,038 Vietnam-era women Veterans who never deployed to Vietnam (n=126). Both the crude (odds ratio [OR]=1.22 [0.96, 1.55]) and adjusted ORs (OR=1.18 [0.91, 1.51] suggested that the odds of developing BC were statistically equivalent between groups (28). Macfarlane and colleagues (2003) followed a cohort of 51,721 Gulf War Veterans and 50,755 matched service personnel and found no increased risk among Veterans that deployed in support of the Gulf War (IRR=0.59 [0.21, 1.62]) (32). Young and colleagues (2010) followed 621,902 Gulf War Veterans and 746,248 non-Gulf War Veteran controls. Gulf War Veterans had a statistically equivalent IR of BC among men (Proportional IR [PIR]=0.78 [0.39, 1.58]) and women (PIR= 1.01 [0.86, 1.20]), respectively (44). Gaffey and colleagues (2023) conducted a cohort study of 576,601 women Veterans (n=141,935 OEF/OIF-deployed). Those who deployed in support of OEF/OIF were 23% [17%, 29%] less likely to be diagnosed with BC (RR=0.77; [0.71, 0.83]) (25).

#### General incidence rates

Four studies provided general incidence rates without a comparison group. Hoiberg & Ernst (27) conducted a cohort study of 364 active-duty Navy women (n=47 cases) and found an IR by age (overall 34.1 per 100,000) (27). Ajene and colleagues (22) conducted a cohort study of 78 women Naval personnel and found a BC IR of 8.53 per 100,000 persons (22). Strand and colleagues (2014) analyzed 268 Norwegian women military peacekeepers deployed to Kosovo, and one incident case of BC was observed (38). Strand and colleagues (2020) reanalyzed their 2014 cohort of Norwegian military peacekeepers deployed to Kosovo: 275 women were included and three BC cases were found (40).

### Volatile organic compounds & endocrine-disrupting chemicals

Two papers measured exposure to VOCs. Rennix and colleagues (2005) conducted a cohort study of 274,596 enlisted Army women. Exposure to VOCs was tied to job titles, categorized as none, low, medium, or high (35). None of the top military occupational specialties were associated with BC risk. However, history of moderate or high exposure to VOCs was associated with a 48% higher incidence (IRR=1.48 [1.03, 2.12]) (35). Ruckart and colleagues (2015) led a case-control study of Marines stationed at the Camp Lejeune garrison who were exposed to contaminated groundwater (71 cases of male BC, 373 controls). Adjusted ORs for high residential cumulative exposures to tetrachloroethylene, t-1,2 dichloroethylene, and vinyl chloride were 1.20 [0.16, 5.89], 1.50 [0.30, 6.11], 1.19 [0.16, 5.89], respectively (36). Ever being stationed at Camp Lejeune and high cumulative exposures to VOCs were associated with an earlier age at onset for male BC, though it was not statistically significant (36).

Carran and Shaw (24) conducted a cohort study of 71 Veterans and 76 female adult children of Veterans. They found an increased risk of BC among female adult children of New Zealand Veterans deployed to Malaya who were exposed to the EDC dibutylphthalate (24).

### Tactile herbicides (agent orange)

One paper directly measured exposure to the tactile herbicide, Agent Orange. Yi & Ohrr (43) mapped each unit’s post location and tactile area of responsibility to known geographic regions of chemically treated areas. Korean Vietnam-era Veterans (n=180,251 with follow-up from 1992-2003) were given an Exposure Opportunity Index and stratified into categories (high vs. low) in a cohort study (43). Exposure to high levels of Agent Orange was not associated with an increased risk of BC (adjusted HR=0.53 [0.12, 2.26]) (43).

### Serum 25-hydroxyvitamin D/ultraviolet B sunlight exposure

Mohr and colleagues (2013) examined the relationship between pre-diagnostic serum 25-hydroxyvitamin D (Vitamin D) and risk of BC among active-duty personnel using a nested case-control study with 600 incident cases and 600 controls. No statistically significant relationship was found between serum Vitamin D levels and odds of BC.

## Discussion

This scoping review covered 28 papers on the relationships between military service, MEE, and BC among active-duty personnel and Veterans. Unfortunately, evidence is still needed before conclusive remarks can be made. Most papers on military service or deployment reported a decreased or statistically equivalent risk of BC, while a few larger surveillance studies found an increased risk. When considering the effects of military service and deployment on risk of BC, individual and environmental risk factors should be considered (49). If risk factors are not controlled for, findings may be biased by the Healthy Soldier Paradox (25).

The Healthy Soldier Paradox occurs when healthier personnel are deployed in support of military operations and sicker personnel are not deployed or are given different military occupations (50). This form of sampling bias can lead to inaccurate associations between deployment and health outcomes, where deployed individuals appear to have a lower risk of adverse outcomes than non-deployed personnel. Recent work has called the Healthy Soldier Paradox into question for OEF/OIF/OND-era Veterans (51), as OEF/OIF/OND-era Veterans have a higher risk of mortality when compared to the general U.S. population. However, it may be that healthy solider effects vary by outcome, such that OEF/OIF/OND-era Veterans may have a higher risk of mortality but a decreased risk of BC (25). In a study of 31,548 military healthcare system users with BC and 63,096 controls with BC, the military healthcare system users had a significantly lower risk of mortality (24% [20%, 29%]) (52). This lower mortality risk was found across all ages, Stages II, III, and IV tumors, and for Black and White patients (52), suggesting that military personnel may also benefit from an equal-access healthcare system (18, 19).

Many studies have few years of follow-up, small sample sizes, lack accurate measurement, and suffer from misclassification bias. *Follow-Up*: Epidemiological analyses of cancer require a sufficiently long follow-up so that cases can occur. Most cases of BC occur in women aged 50+ (6), and many personnel from post-9/11 conflicts may not be in this age group yet. *Small Sample Size*: Women personnel and Veterans with MEE concerns are still a relatively small group from an epidemiological perspective. *Accurate Measurement of Exposure*: Investigators often rely on participant recall for MEE (leading to recall bias) and do not consider that most MEE are transient, may occur more than once, and may occur with varying severity. *Accurate Measurement of Outcome*: Measurement of BC has improved in recent decades due to advances in mammography screening (53) and modern classification codes (e.g., ICD-10). However, characteristics of the breast tumor (e.g., T-stage, N-stage, M-stage) and histological and molecular subtyping are not often explored. *Misclassification Bias*: Misclassification bias reflects an issue with categorizing participants by exposure/outcome status. Without accurately measuring MEE, participants may be misclassified, leading to null results (54). For many articles, exposures are generalized to entire groups, but individual-level data are needed.

Most papers in this review considered military service or military deployment as an exposure when measuring BC risk in personnel. Unlike most social and environmental exposures, military service (yes/no) is not well operationalized and leaves a lot to be desired in terms of specificity. Characteristics of military service (e.g., military occupation and job duties, deployment location, rank, military branch, number of years served, and MEE) should be measured in future studies, as these factors may improve our detection of the Healthy Soldier Paradox. Additionally, tying specific military occupations and job duties to MEE will be crucial for determining causality, and will inform policy and practice regarding the expansion of personal protective equipment and environmental toxin passive monitoring devices in the field.

With these limitations stated, several conclusions may still be found. Exposure to VOCs appears to impact the downstream risk of BC among military personnel (35, 36). Specific VOCs’ effects are largely unknown, but the risk of BC appears stronger among women than among men, and the mechanism of action may be through oxidative damage, cytotoxicity, and genotoxicity (55–57). The effects of EDCs have not been sufficiently studied in military samples, but they are known to impact risk of BC in civilians (58). Vitamin D (34, 59, 60) may not be considered an environmental exposure, but ionizing radiation from the sun would be an important MEE. Future work should measure UV exposure, heat, and drought directly. No papers directly assessed the effects of AHOBP or depleted uranium, but many Veterans who deployed in support of Gulf War and OEF/OIF/OND with MEE to AHOBP and depleted uranium are now at an age where BC is a salient concern (61). One large study looked at Agent Orange and BC risk and did not find an association. Unfortunately, no studies were found on other tactile herbicides (e.g., Agents White, Blue, Purple, Pink, Green) or pesticides. Many MEE included in this review were not specific to military populations. VOCs, EDCs, and carcinogenic airborne hazards are well-known occupational exposures in the civilian sector. Too little work has been done to understand if the effects of these generic exposures are moderated by military service. Finally, it is important to recognize that a greater number of high-quality articles will be needed to draw significant conclusions that link MEE and BC, as surveillance cohort studies are insufficient to draw causal links.

### Recommendations

Future studies should: 1) Measure MEE in real time (e.g., dose, duration, source, route of entry) using ecological momentary assessment or passive monitoring; 2) Study specific VOCs, EDCs, and AHOBP; 3) Compare deployed to non-deployed military personnel and include a group of civilian controls when possible; 4) Recruit a diverse group of women and gender-diverse personnel, including all military branches, races/ethnicities, ranks, occupations, and deployment locations; 5) Determine warfare theater/era effects; 6) Measure BC histological/molecular subtypes; 7) Expand years of follow-up and increase recruitment; and 8) Explore biological plausibility by tying MEE to specific carcinogenic pathways.

Findings on MEE and BC are varied, in part due to the Healthy Soldier Paradox, potential misclassification of exposure(s), and modest sample sizes. The strongest evidence with reproducible findings appears to be Veterans’ increased risk of BC after being exposed to VOCs.

## Author contributions

DJ: Writing – review & editing, Writing – original draft, Supervision, Software, Project administration, Methodology, Investigation, Formal analysis, Data curation, Conceptualization. MA: Writing – review & editing, Writing – original draft, Supervision, Software, Project administration, Investigation, Formal analysis, Data curation. DG: Writing – review & editing, Writing – original draft, Software, Investigation, Formal analysis, Data curation. AI: Writing – review & editing, Writing – original draft, Software, Investigation, Formal analysis, Data curation. JJ: Writing – review & editing, Writing – original draft, Supervision, Resources, Project administration, Funding acquisition. MA: Writing – review & editing, Writing – original draft, Supervision, Resources, Project administration, Funding acquisition.
